# Thyroid and renal cancers: A bidirectional association

**DOI:** 10.3389/fonc.2022.951976

**Published:** 2022-09-23

**Authors:** Maria Irene Bellini, Eleonora Lori, Flavio Forte, Augusto Lauro, Domenico Tripodi, Maria Ida Amabile, Vito Cantisani, Marzia Varanese, Iulia Catalina Ferent, Enke Baldini, Salvatore Ulisse, Vito D’Andrea, Daniele Pironi, Salvatore Sorrenti

**Affiliations:** ^1^ Department of Surgical Sciences, Sapienza University of Rome, Rome, Italy; ^2^ Department of Urology, M. G. Vannini Hospital, Rome, Italy; ^3^ Department of Radiological, Anatomopathological and Oncological Sciences, Sapienza University of Rome, Rome, Italy

**Keywords:** thyroid cancer, renal cancer, multiple cancer, cancer surveillance and screening, cancer risk

## Abstract

There is a deep interrelation between the thyroid gland and the kidney parenchyma, with dysfunction of the first leading to significant changes in renal metabolism and *vice versa*. Given the recognition of cancer as a systemic disease, the raise of thyroid tumors and the common association of several malignancies, such as breast cancer, prostate cancer, colorectal cancer, and other, with an increased risk of kidney disease, public health alert for these conditions is warranted. A systematic review of the current evidence on the bidirectional relationship between thyroid and renal cancers was conducted including 18 studies, highlighting patient’s characteristics, histology, time for secondary malignancy to develop from the first diagnosis, treatment, and follow-up. A total of 776 patients were identified; median age was 64 years (range: 7–76 years). Obesity and family history were identified as the most common risk factors, and genetic susceptibility was suggested with a potential strong association with Cowden syndrome. Controversy on chemo and radiotherapy effects was found, as not all patients were previously exposed to these treatments. Men were more likely to develop kidney cancer after a primary thyroid malignancy, with 423/776 (54%) experiencing renal disease secondarily. Median time after the first malignancy was 5.2 years (range: 0–20 years). With the advancement of current oncological therapy, the prognosis for thyroid cancer patients has improved, although there has been a corresponding rise in the incidence of multiple secondary malignancy within the same population, particularly concerning the kidney. Surgery can achieve disease-free survival, if surveillance follow-up allows for an early localized form, where radical treatment is recommended.

## Introduction

Thyroid cancer (TC) is one of the most rapidly increasing malignancies in Western countries, with an annual incidence rate of 5.4% in men and 6.5% in women (1). Much of this rise is largely due to early detection using more sensitive diagnostic procedures, including Artificial Intelligence, performed for other medical reasons and able to identify incidental small thyroid nodules, otherwise missed (2–4). Certain risk factors for TC are female sex, family history of TC, radiation exposure, lymphocytic thyroiditis, and reduced iodine intake (5, 6). On the basis of the histological and the clinical behavior, TCs are divided into well differentiated and poorly differentiated; well-differentiated TCs include the papillary and follicular histotypes (7). Surgery, either lobectomy or total thyroidectomy, represents the standard therapeutic approach for well-differentiated TC; radioactive iodine therapy is recommended for high-risk patients (5). Ablation and active surveillance are of increasing importance in patients who refuse surgery or are unfit for.

Improvements in the detection of TC and therapeutic strategies have likewise resulted in a more favorable course for this disease. Because the mortality rates for TC remained stable at around 0.5 deaths per 100,000, the number of patients surviving is on the rise (8, 9).

On the other hand, renal cancer, or renal cell carcinoma (RCC), is the 9^th^ common cancer in men and the 14^th^ one in women. RCC frequently presents incidentally; in fact, it is asymptomatic in most cases. Therefore, the diagnosis of patients with localized renal cancer, which is potentially treatable only with surgery or ablation, is almost always accidental (10). Identified risk factors include male sex, smoking tobacco, obesity, and hypertension (10, 11). RCC comprises an heterogeneous group of histological subtypes: Clear cell renal cell carcinoma (ccRCC), papillary, and chromophobe are the most common solid RCC (11). Nephron-sparing surgery or partial nephrectomy has evolved as the standard of care in patients with localized RCC; ablation and active surveillance are traditionally alternatives for patients who are unfit for surgery (11).

Thyroid interrelation with the kidney is well known (12); on the one hand, this gland is necessary for renal cells growth and for the maintenance of hydro-electrolyte homeostasis; on the other hand, the kidney eliminates thyroid hormones and regulates their serum level. There is therefore a deep interrelation among the two organs, with thyroid dysfunction causing significant changes to renal metabolism and *vice versa (* 13).

Cancer is a systemic disease, and many common cancers, such as breast cancer, prostate cancer, colorectal cancer, and other, are associated with an increased risk of kidney cancer development, especially within the first 5 years after their diagnosis (14). Because the risk of second cancers after the diagnosis of primary TC is elevated (15), too, the aim of this manuscript is to review the current state of knowledge on the interrelationship between thyroid and renal cancers.

## Methods

**Figure 1:**
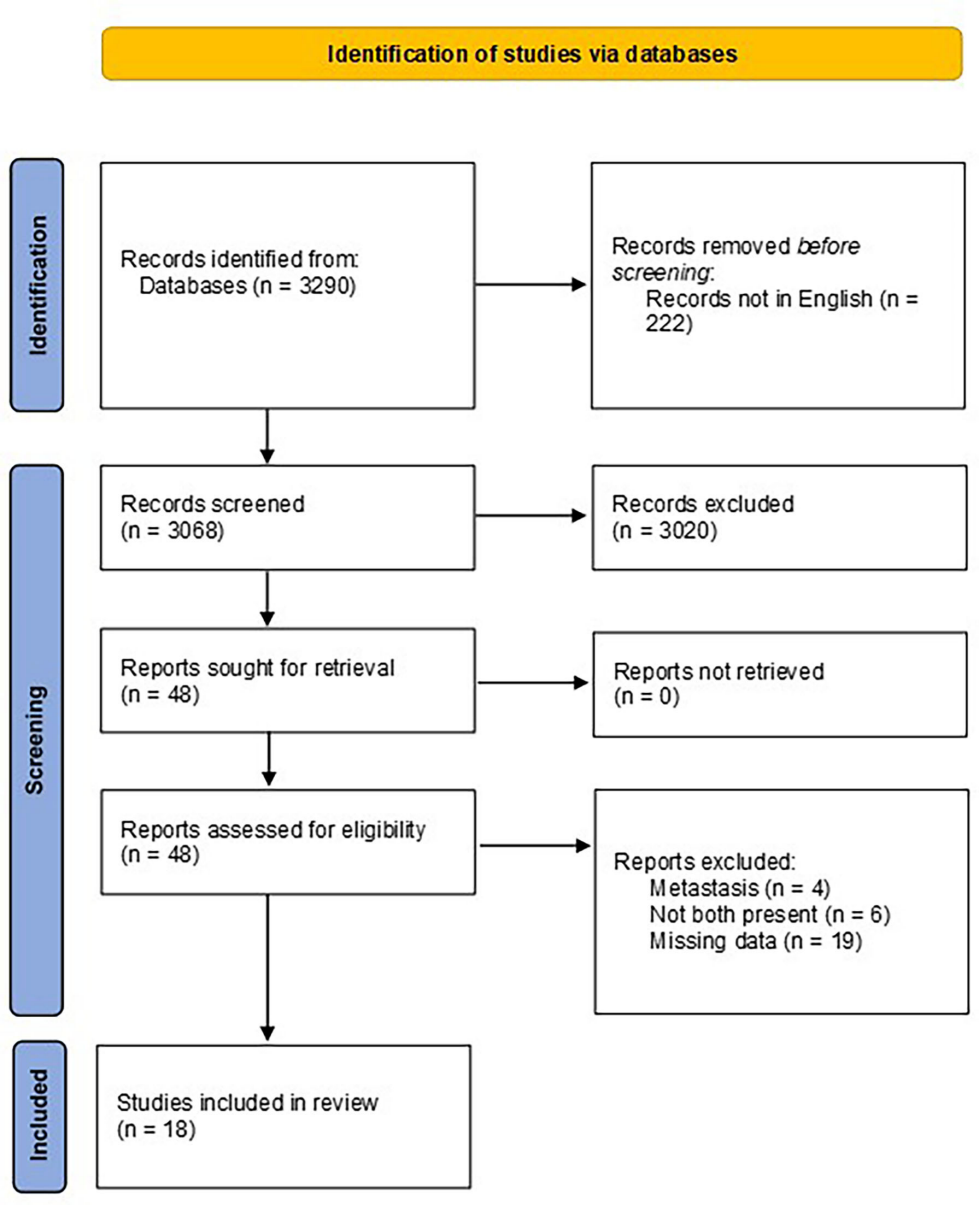
PRISMA diagram.

This review was conducted in accordance with the Preferred Reporting Items for Systematic Reviews and Meta-Analyses Statement (PRISMA) (16). The search was run in February 2022 across PubMed, Web of Science, and Scopus databases and was restricted to articles written in English only. References were cross-checked for additional relevant studies. The retrieved lists were exported to a reference manager (EndNote™) to eliminate duplicates, as shown in **Figure 1**.

Keywords “thyroid cancer” and “renal cancer” were used to include studies evaluating TC characteristics in patients previously affected by kidney cancer or kidney cancer characteristics in patients previously affected by TC. Reports of metastases were excluded from the analysis.

The research was performed by two independent investigators; subsequently, the results were compared and combined; in case of disagreement on the value of the selected papers, an additional comparison was crucial in the decision-making process. Only published literature was included, and no date limits have been set. Only English language articles were included; reviews, editorials, and repeated or redundant manuscripts were excluded. Only registry analyses and retrospective studies, mostly case reports, were found and included in the present review.

Data extraction was performed thereafter, including the details of title, authors, date of publication, country, research design, patients’ characteristics, and outcomes.

A risk of bias assessment was performed using the Newcastle–Ottawa Scale quality assessment star system (**Table 1**), in which a paper is judged on the selection of the study groups, the comparability of the groups, and the ascertainment of either the exposure or the outcome of interest for case control or cohort studies, respectively (24).

**Table 1:** Newcastle–Ottawa Scale (NOS) quality assessment star system.

NOS^17^										
COHORT STUDY										
Article	Selection				Comparability	Outcomes			Total	
	Selection of nonexposed cohort	Representativeness of exposed cohort	Ascertainment of exposure	Outcome not present at the start of the study		Assessment of outcomes	Length of follow-up	Adequacy of follow-up	
Canchola et al. (17)	☆	☆	☆	☆	☆☆	☆	☆	☆	**9/9**
CASE CONTROL										
**Article**	**Selection**				**Comparability**	**Outcomes**			**Total**	
	**Case Definition**	**Representativeness of the Cases**	**Selection of Controls**	**Definition of Controls**		**Ascertainment of Exposure**	**Non-Response Rate**	**Same methods of** **Ascertainment for cases and control**	
Abdel-Rahman, et al. (14)	☆	☆	☆	☆	☆	☆	☆	☆	**8/9**
Antonelli, et al. (18)	☆	☆	☆	☆	☆☆	☆	☆	☆	**9/9**
Carhill et al. (19)	☆	☆	☆	☆	☆☆	☆	☆	☆	**9/9**
Murray, et al. (20)	☆	☆	☆	☆	☆☆	☆	☆	☆	**9/9**
Murray S, et al. (21)	☆	☆	☆	☆	☆	☆	☆	☆	**8/9**
Ngeow, et al. (22)	☆	☆	☆	☆	☆☆	☆	☆	☆	**9/9**
Van Fossen, et al. (23)	☆	–	☆	☆	☆	☆	☆	☆	**7/9**

The stars mean the grading according to the Newcastle-Ottawa scale.

## Results

A total of 3,290 manuscripts were retrieved from the search; following exclusion based on title and abstract screening (*n* = 3,020) and after full text read (*n* = 48), the remaining studies included in the review were 18 (**Table 2**). The majority (11/18) were case reports. A total of 776/64,187 patients were identified.

**Table 2:** Results.

Article	Year	Type of study	Case	Sex	Age	Histology	Genetic syndrome	Risk factors	Thyroid cancer	Interval to second cancer	Conclusion
Abdel-Rahman (14)	2017, Egypt	Case control	341/9861	N/A	N/A	N/A	N/A	Treatment factors (radiation) Common etiology factors (smoking) Rare hereditary cancer syndromes	Primary	5 years	Beyond 5 years, patients with primary thyroid cancer have an enhanced risk to develop a second primary kidney cancer. This link may be an expression of a particular genetic makeup determining patients’ susceptibility to both cancers.
Albores-Saavedra, et al. (25)	2014, Mexico	Case report	2/2	F	72 54	Papillary urothelial carcinoma and PTC	N/A	No specific risk factors were identified.	Second Second	14 years 1,5 years	These malignant neoplasms do not apparently share similar risk factors.
Antonelli, et al. (18)	2012, Italy	Case control	15/285	N/A	N/A	N/A	N/A	No specific risk factors were identified.	Second	N/A	The risk of development of a second neoplasia in patients with RCC increases with aging.
Canchola et al. (17)	2005, USA	Cohort study	16/10932	F	55	PTC and RCC not otherwise specified	N/A	Obesity increases the risk of both thyroid and kidney cancer	Primary	3 years	Increased surveillance is warranted for kidney cancer among women with thyroid cancer.
Carhill et al. (19)	2014, USA	Case control	117/23514	N/A	N/A	Papillary thyroid carcinoma (85%) and ccRCC (79%)	N/A	Genetic susceptibility, implication of clinical therapy	N/A	6 years	The association between thyroid and kidney cancer needs further investigation.
Oh, et al. (26)	2015, Korea	Case report	1/1	M	50	ccRCC and PTC	N/A	Family history of thyroid cancer	Synchronous	0	No specific risk factor or genetic syndrome were identified.
Atta, et al. (27)	2016, Egypt	Case report	1/1	F	76	ccRCC and PTC	No mutations were detected	Family history of colon, lung, kidney and thyroid cancer.	Primary	14 years	No genetic mutation was detected, despite the family history.
Kim, et al. (15)	2020, Canada	Case report	1/2	M	22	Chromophobe RCC and PTC	Cowden syndrome (CS)	Family history of kidney and thyroid cancer.	Primary	12 years	Thyroid neoplasia and RCC are minor diagnostic criteria for CS.
Klain, et al. (28)	2021, Italy	Case report	1/2	M	64	ccRCC and PTC (tall-cell variant)	N/A	No specific risk factors were identified	Second	20 years	No specific risk factor or genetic syndrome were identified.
Ma, et al. (29)	2014, China	Case report	1/1	F	35	ccRCC+ SFT and PTC + follicular thyroid carcinoma	N/A	Negative family history of neoplasia	Synchronous	0	No specific risk factor or genetic syndrome were identified.
Malchoff et al. (30)	1999, USA	Family report	31/31	N/A	N/A	Papillary renal carcinoma and PTC	Mutation of a gene that maps to 1q21	No specific risk factors, except for genetics, were identified	N/A	N/A	Familial association of PTC with papillary renal neoplasia defines a distinct familial tumor syndrome.
Murray, et al. (20)	2016, USA	Case control	12/3066	6 F 6 M	53 64	PTC and RCC not otherwise specified	N/A	No specific risk factors were identified	Second	N/A 7 years	The rate of thyroid cancer in both women and men surgically treated for RCC was significantly higher. Observed association is unlikely due to treatments effects because primary treatment in renal cancer is surgical.
Murray S, et al. (21)	2013, USA	Case control	3/433	N/A	N/A	N/A	N/A	Older and radiation exposure	Synchronous	0	Papillary thyroid cancer is the most frequent histologic type associated to RCC.
Ngeow, et al. (22)	2014, USA	Case control	2/114	M F	7 36	N/A	PHTS	PTEN mutation	Primary	14 8	A bidirectional association between thyroid and renal cancers suggests shared genetic and environmental risk factors.
Peng, et al. (31)	2019, China	Case report	1/1	M	58	ccRCC and micro-papillary thyroid carcinoma	N/A	No specific risk factors were identified	Primary	1 years	Integrin ανβ6 is positively expressed in multiple primary cancer, also in patients with RCC and thyroid cancer.
Samarasinghe, et al. (32)	2020, USA	Case report	1/1	F	56	ccRCC and PTC + medullary thyroid cancer	RET mutational analysis was negative	Family history of breast cancer and RCC	Primary	2 years	RET mutational analysis was negative.
Song, et al. (33)	2017, Canada	Case report	1/1	M	72	ccRCC and PTC	N/A	N/A	Synchronous	0	Tumour-to-tumour metastasis of a thyroid cancer into a primary renal neoplasm is extremely rare and maybe resulting from rich vascularity and perfusion to enable successful delivery and deposition of metastatic tumour cells.
Van Fossen, et al. (23)	2013, USA	Case control	230/15940	90 M 60 F	N/A	N/A	N/A	N/A	60 primary 80 second	N/A	This study demonstrated a bidirectional association between thyroid and renal cancers. This association is more likely explained by shared genetic and environmental factors.

ccRCC, clear cell renal cell carcinoma; PTC, papillary thyroid carcinoma; N/A, not available; F, female; M, male.

## Patients’ characteristics

Median age was 64 years (range: 7–76 years). The association of thyroid and renal malignancies was more often observed in the male population. After evaluating the time between the two malignancies for all the studies included in the review, in no case, a significant difference was detected: The median interval between first and second cancer was 5.2 years (range: 0–20 years).

Most patients presented a TC as first primary malignancy (423 out of 776; 54%), and 110 patients (14%) developed a TC as second primary malignancy; a renal tumor was synchronous in only six patients out of 776 (0.78%).

## Histopathological characteristics

With regards to histology, the papillary phenotype of TC was found in all patients. Sporadic cases of follicular carcinoma (29) and medullary (32) thyroid carcinoma have also been identified; however, both of them were also associated with a papillary thyroid carcinoma. On the contrary, with regards to renal carcinoma, a greater variety was observed concerning histology. In the series evaluated, the most represented type was ccRCC; however, sporadic cases of other renal malignancy cases were also reported, namely, urothelial (25), chromophobic (34), and papillary renal carcinoma (30).

## Sex

With regard to TC, female sex was universally identified as a risk factor (17, 20); on the contrary, male sex is associated to the development of RCC (17). From the report by Van Fossen et al. (23), female TC patients had a twofold increase in the prevalence of a subsequent renal cell cancer (23), and female renal cell cancer patients had a 1.5-fold increase in the prevalence of TC; male patients with TC had 4.5-fold prevalence increase of subsequent RCC, and male patients with RCC had an increased threefold prevalence of subsequent TC. Male sex emerged as a risk factor of association between thyroid and kidney cancers (18).

## Common identified risk factors

**Figure 2:**
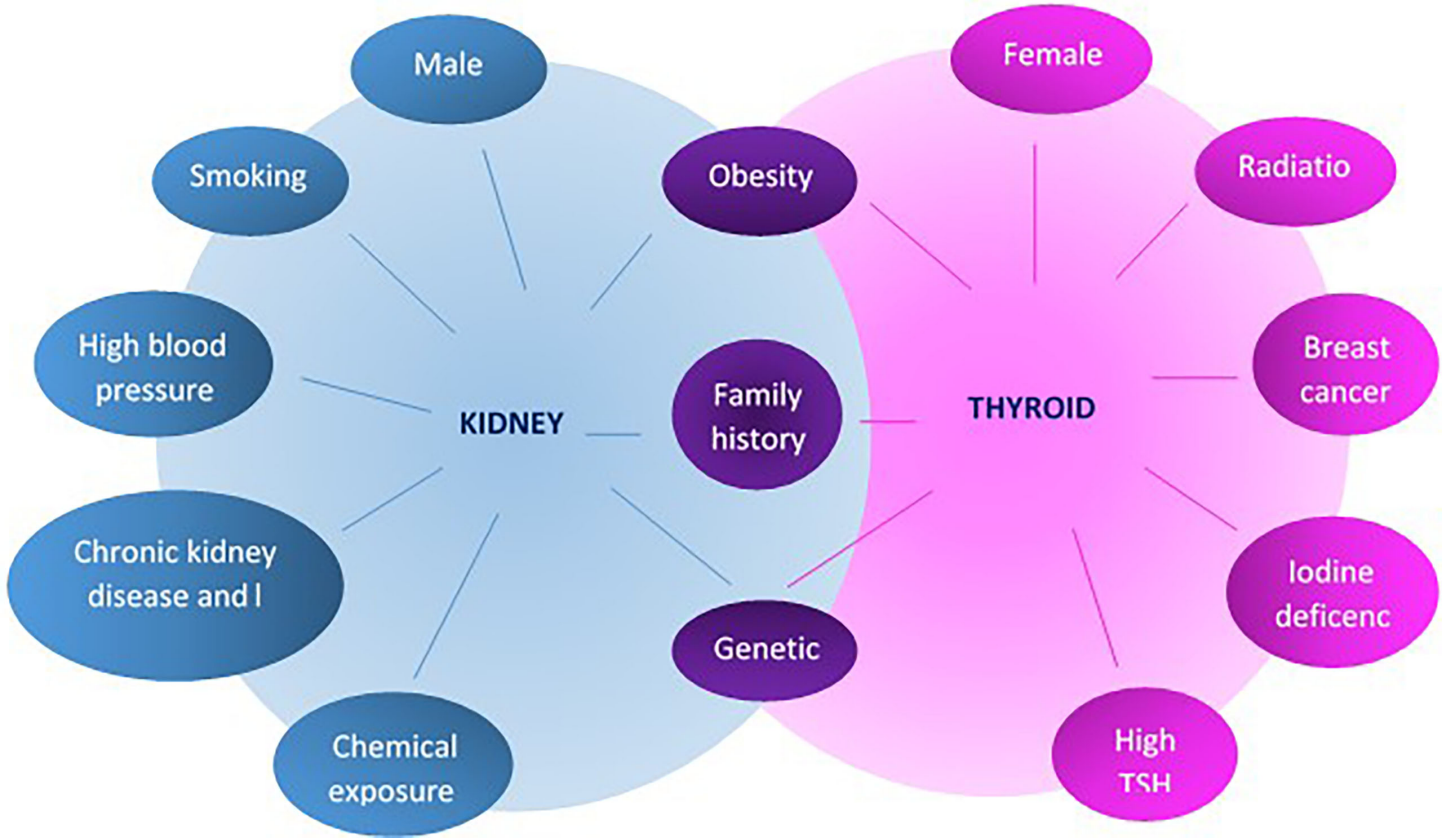
Risk factors for the development of thyroid and kidney cancer.

Identified common risk factors between thyroid and kidney cancers are few (19, 25), with obesity remaining a unique denominator to develop malignancy in general and in particular for these two; see **Figure 2**. Family history (29) of both cancers is also well recognized, and as previously mentioned, male sex as well as aging increased the risk to develop both cancers and, in particular, RCC (18).

Although radiation exposure is a known risk factor for the development of neoplasms and, in particular, for TC, no correlation between radiotherapy and the development of both TC and renal cancer was identified in any of the studies included in the review. Murray et al. (21) observed, in fact, that only 35% of patients with TC and an additional primary cancer, whether it is RCC or not, reported radiation exposure in their medical history. Given the recognized increased risk of TC following other primary malignancies, particularly RCC, it seems unlikely that the increased incidence could be due to the carcinogenic effect of radiations (14).

Although many chemotherapeutic agents are known to be carcinogenic, the patients have not undergone chemotherapy, and it is therefore not possible to evaluate the carcinogenic effects of these drugs.

## Genetic syndrome

TC is associated with a heterogeneous pattern of genetic mutations involving the mitogen-activated protein kinase (MAPK) pathway (6). The main genetic mutations are represented by the oncogenes RAS and BRAF; in particular, BRAF^V660E^ is present in about half of the PTCs (35).

Excluding the familial forms of medullary thyroid carcinoma, such as familial medullary thyroid carcinomas and multiple endocrine neoplasia (MEN), the familial forms of thyroid carcinoma are numerous and can be divided into syndromes with a prevalence of non-thyroid neoplasms and syndromes with a prevalence of TC. The first group includes also familial adenomatous polyposis, Cowden syndrome (CS), Werner syndrome, Carney complex, and Pendred syndrome; the second group includes pure familial papillary thyroid carcinoma with or without oxyphilia and familial papillary thyroid carcinoma with papillary RCC or with multinodular goiter (36).

There are also numerous genetic alterations involved in the development of RCC, in particular, the most important mutations involving the tumor-suppressor Von Hippel-Lindau (VHL), observed in about 80% of ccRCC (9). Hereditary forms of RCC include von Hippel-Lindau syndrome, hereditary papillary RCC, Birt-Hogg-Dubé syndrome, hereditary leiomyomatosis, and tuberous sclerosis (11).

The link between thyroid and kidney cancer may be an expression of genetic makeup that increases patients’ susceptibility to both malignancies (26, 27). Although this condition seems the most likely to occur, few studies have identified genetic syndromes or specific mutations, which are associated with the increased susceptibility to these two tumors, highlighting a familiar link (23, 32).

In the present review, CS (15, 22) was confirmed a risk factor leading to thyroid and renal cancers. CS is part of the Phosphatase and tensin homolog (PTEN)-hamartoma tumor syndrome, a disorder caused by a germline mutation of PTEN, a tumor suppressor gene. This syndrome is associated with the development of a variety of tumors, both benign and malignant (11): Thyroid carcinoma is one of major diagnostic criteria for CS, whereas RCC is part of the minor criteria.

PTEN mutation, even in the absence of the CS, was identified as risk factor (22), with Integrin ανβ6 positively expressed in multiple primary cancer, among which TC and RCC (31). The genetic mutation 1q21 (30) was identified in forms where PTC was associated to papillary renal neoplasia tumors, highlighting in this way a peculiar familial tumor syndrome (**Table 2**).

## Time of second cancer occurrence

According to the analysis of the data presented in **Table 2**, the median interval between first and second cancer was 5.2 years (range: 0–20 years), with no substantial difference in the time interval considering one or the other cancer as the first presented.

## Treatment and follow-up

For both thyroid and kidney cancers, treatment of choice was represented by surgical excision (28), with no differences if they were primary, synchronous, or second malignancies. In most cases, the selected patients were affected by localized neoplasms; thus, no need for systemic therapy was required. Furthermore, as they were detected at an early stage, surgery had a curative effect. In case of synchronous malignancy, radical nephrectomy first and then total thyroidectomy with lymphoadenectomy were carried out (33).

In general, management of PTC remained equivalent, regardless of whether or not the patient had a synchronous or antecedent non-thyroidal neoplasia (21).

In most of the reported cases, the second cancer was identified during follow-up, except for the few cases of synchronous tumors, for which the pre-operative investigations made it possible to identify the second neoplasm at an earlier stage (33). In consideration of the increased risk of developing a second tumor after the primary cancer, all the authors recommended to keep this risk in mind during the follow-up of thyroid and kidney malignancies.

## Discussion

The present review evaluated the association between thyroid and renal cancers, regardless of which cancer occurred first, highlighting that each primary thyroid or renal malignancy increases the relative risk of subsequent malignancy in the remnant organ of the survived patients. This applies to both sexes, particularly relevant in men (23), even if other reports document an increase only in treated female TCs (37).

Although there is a risk of a second primary tumor following primary invasive neoplasms and, specifically, there is a reciprocal association between thyroid and renal cancers, the estimated risk for the development of both cancers is low, with an incidence of about 1% according to Van Fossen et al. (23) For this reason, it is not considered necessary to include diagnostic screening tests in the follow-up of these neoplasms, compared with what is already foreseen for general population. If a more targeted preventive screening is deemed appropriate (7, 38, 39) in the presence of additional risk factors, ultrasound scans of the neck and kidneys may be indicated.

A bidirectional association between thyroid and renal cancers can be explained by shared genetic and/or common environmental risk factors including recognized etiological factors (i.e., smoking and obesity), or rare genetic syndromes predisposing to both events and regardless of the use of any forms of radiation treatment (14). Furthermore, individuals who develop both thyroid and renal carcinomas may represent a unique subset of cancer patients (19).

TC is associated with a number of genetic mutations leading to a different aggressive behavior. BRAF and RAS rearrangements remain the principal oncogenes, although other mutations, namely, TERT promoter and in TP53, as well as PIK3CA–PTEN–AKT–mTOR pathway and SWI–SNG complex (40), synergistically concur to worse outcomes and can be used in tumor prognostication (41). In the case of medullary carcinoma, RET mutation is commonly identified, supporting a distinct clonal origin in the case of a coexisting papillary tumor, as different cellular types might be affected simultaneously (42, 43).

The majority of renal carcinomas are sporadic, and numerous are the genetic alterations involved; in particular, the most important mutations involve the tumor-suppressor VHL, observed in about 80% of ccRCC (9). A genetic predisposition accounts for around 4% of the incidence of this malignancy, namely, in people affected by von Hippel-Lindau disease, hereditary papillary renal cancer, hereditary leiomyomatosis and renal cancer, and Birt-Hogg-Dubé syndrome. Other studies have also proposed possible genetic correlations between thyroid and renal cancers; Malchoff et al. (30) identified a distinct familial tumor syndrome linked to a germline mutation in chromosome 1q21 and characterized by a familial association of papillary TC, nodular thyroid disease, and papillary renal neoplasia. TC of follicular origin and renal cancer have also been found with greater frequency in CS, a hereditary cancer syndrome associated with a germline mutation in PTEN (44) and characterized by the presence of multiple hamartoma and dermatologic manifestations such as acral keratosis and facial trichilemmomas. For CS, thyroid carcinoma is one of the major diagnostic criteria, whereas RCC is part of the minor criteria.

Interestingly, as our review reported, the phenomenon of increased genetic instability and reduction of tumor immunity in multiple cancer patients was confirmed by the case of the woman with medullary, papillary, and RCC (32), a very rare combination, where even if the patient had no previous endocrine history, her mother was affected by breast cancer, another disease deeply connected to TC (4, 45–47) and the brother presented with RCC, too. A triple malignant tumor was also reported in a male of the same age with thyroid, kidney, and colon being affected (31), demonstrating common expression paths with integrin avß6 in multiple primary cancer (29).

Treatment of choice is surgery, regardless of whether or not the patient had a synchronous or antecedent neoplasia (21). The prognosis is in relation to the biological characteristics of each cancer, with the extent of the surgical procedure aiming to radically excise the mass and potentially reduce the incidence of subsequent cancers (48), without additional chemo and/or radiotherapy. If the patient is unfit for surgery, ablation *via* interventional radiology might represent a valid alternative (14).

Although radiation exposure is a known risk factor for the development of neoplasms and in particular for TC, and many chemotherapeutic agents are known to be carcinogenic, no correlation between radiotherapy or chemotherapy and the development of both TC and renal cancer was identified in any of the studies included in the review. This is mainly because the treatment of localized kidney cancer is primarily surgical, such as the treatment of well-differentiated TC; in fact, the association between kidney and TC is probably not related to radio and chemotherapy treatments (22) but rather to a shared genetic makeup or other environmental factors.

Considering the low risk of developing kidney cancer after TC and *vice versa*, it does not seem necessary to change the follow-up of patients with one of the two cancers to monitor the onset of the other one; however, it is important to always keep in mind that there is an increased risk of developing a second malignancy. It is always necessary to remember that, in subjects with a genetic syndrome that increases the risk of developing tumors, the follow-up should be structured taking into account the underlying genetic pathology.

## Limitations

In consideration of the practical difficulties associated with evaluating the research question in prospective settings, only registry analyses and retrospective studies, mostly case reports, were included in the present review, limiting the evidence achieved. High-quality population databases are recommended with prospective analysis to elucidate on the bidirectional association between thyroid and kidney cancers.

## Conclusions

As for TC, the advancement of diagnostic methods has led to an early treatment and an improvement in prognosis; in the same way, for kidney cancer, the increase in the diagnosis of neoplasms in the early stages has led to an increased survival; therefore, there has been a corresponding rise in the incidence of multiple primary cancers. A bidirectional association between thyroid and renal cancers has been identified and can be explained by shared genetic and common environmental risk factors. Even if there is an association, the coexistence of primary thyroid and RCC is rare. The standard treatment for both thyroid and kidney cancers remains surgery, which does not need to be associated with adjuvant therapies in the early stages, and the follow-up does not require special attention from clinicians or screening tests, except in cases of known genetic syndromes.

## Data availability statement

The original contributions presented in the study are included in the article. Further inquiries can be directed to the corresponding author.

## Author contributions

Conceptualization: MB, EL, SS and DP. Methodology: MB, SS, FF, AL, DT, MA and MV. Investigation and Data curation: EL, DP, VC, VD, EB and SU. Writing—original draft preparation: MB and EL. Writing—review and editing: MB, EL, SS, DP, FF, AL, DT, MA, MV, VC, EB and SU. Supervision: SS, DP, FF, SU and AL. All authors contributed to the article and approved the submitted version.

## Conflict of interest

The authors declare that the research was conducted in the absence of any commercial or financial relationships that could be construed as a potential conflict of interest.

## Publisher’s note

All claims expressed in this article are solely those of the authors and do not necessarily represent those of their affiliated organizations, or those of the publisher, the editors and the reviewers. Any product that may be evaluated in this article, or claim that may be made by its manufacturer, is not guaranteed or endorsed by the publisher.

## Abbreviations

ccRCC, clear cell renal cell carcinoma; CS, Cowden syndrome; PTC, papillary carcinoma of the thyroid; RCC, renal cell carcinoma; TC, thyroid cancer.
